# Status of visual impairment among indigenous (Orang Asli) school children in Malaysia

**DOI:** 10.1186/s12889-019-6865-3

**Published:** 2019-06-13

**Authors:** Rokiah Omar, Wan Mohd Hafidz Wan Abdul, Victor Feizal Knight

**Affiliations:** 1 Optometry & Vision Science Program, Faculty of Health Sciences, Universiti Kebangsaan Malaysia, Kuala Lumpur, Malaysia; 2 University Community Transformation Centre, Universiti Kebangsaan Malaysia, Bangi, Selangor Malaysia; 3 Department of Ophthalmology Hospital Tuanku Jaafar, Seremban, Negeri Sembilan Malaysia; 4 Faculty of Medicine and Defence Health, National Defence University of Malaysia, Sungai Besi Campus, Kuala Lumpur, Malaysia

**Keywords:** Orang Asli, School children, Hyperopia, Vision screening, Near work

## Abstract

**Background:**

School children are considered a high-risk group for visual impairment because uncorrected refractive errors and problems such as amblyopia can seriously affect their learning abilities and their physical and mental development. There are many studies reporting the prevalence of refractive errors among school children of different ethnic groups in Malaysia, however, studies concerning the prevalence of refractive errors among indigenous or Orang Asli children are very limited. Therefore, the objective of this study was to determine the prevalence and causes of visual impairment among Orang Asli children.

**Methods:**

One hundred ten Orang Asli children aged 7 to 12 years old in Negeri Sembilan, Malaysia were selected. 51% of these children were boys while the remainders were girls. They underwent visual acuity test, cover test, Hirschberg’s test, ocular external assessment and ophthalmoscopy. Children who failed the vision screening were then referred for further eye examination.

**Results:**

Of these 110 Orang Asli children, 46 failed the vision screening and subsequently 45 of them were confirmed to have visual problems (40.9% of the total subjects). The main cause of visual impairment in this study was refractive error (34.5% of the total subjects) where the main refractive error found was hyperopia (28.2%) followed by amblyopia (2.7%), strabismus (1.8%) and ocular abnormalities (1.8%).

**Conclusion:**

Hence, vision screening and a comprehensive eye examination is very important and needs to be done on all Orang Asli children so that any visual problems can be detect at an early stage to avoid the development of learning difficulties among these already disadvantaged children.

## Background

All citizens of Malaysia are entitled to comprehensive health services, including the hardcore poor. According to the official statistics from the Department of Statistics, Malaysia (2010), 76.9% of the Orang Asli (indigenous) population live below the poverty line and 35.2% of the Orang Asli in Malaysia are categorized as hardcore poor [1]. The Orang Asli living in Peninsular Malaysia comprise less than 1% of the total population of Malaysia. The Orang Asli population generally consist of a number of ethnicities which include Negrito, Temuan, Senoi and Proto-Malay [2–5]. Even though they form a fairly small population, comprehensive health services have been available for Orang Asli for more than 60 years, but vision care services are either limited or may be lacking in the various Orang Asli communities.

A previous study has reported that about 90% of people with visual impairment in the world live in countries that are developing and the majority of them live in the interior of their countries [6]. In Australia, the prevalence of vision problems are four to seven times higher among the Aboriginal communities than the non-Aboriginal communities [7, 8]. So far, literature on the visual status or profile of Orang Asli in Malaysia is very limited. The only study conducted on the visual status of Malaysian Orang Asli was conducted by Norlaila et al., (2002) [9]. They found that the visual status was in the normal range except for those who are aged 41 years and above who were found to have presbyopia. The causes of refractive errors found in that study was nearsightedness or hyperopia [9]. Unfortunately, only a small number of children participated in the study. Hence a more detailed study needs to be carried out to identify the visual profile status of Orang Asli children in Peninsular Malaysia. Recent studies have shown that visual impairment can cause disruption to learning and to a decline in academic achievement [10–13]. Hence, early detection through vision screening is essential for identifying visual impairments in children [13, 14], especially the Orang Asli. This is important because 80% of the information received in the learning process is through a vision with other sensory integration [15]. Early intervention of visual impairment will provide clear vision for the children, help them with learning, and further improve their academic achievement and quality of life for their future [13, 14, 16–18].

School children are considered a high-risk group for visual impairment because uncorrected refractive errors can seriously affect their learning abilities as well as their physical and mental development. There are many studies reporting the prevalence of refractive errors among school children of different ethnic groups in Malaysia, however, studies concerning the prevalence of refractive errors among indigenous or Orang Asli children are very limited. Therefore, the objective of this study was to determine the prevalence and causes of visual impairment among Orang Asli children.

## Methods

A sample size of 110 Orang Asli school children aged 7 to 12 years old was determined using Cochran’s sample size formula for categorical data [19]. These children were randomly selected from 154 children who attended Sekolah Kebangsaan Kampung Chenah and Sekolah Kebangsaan Putra in Negeri Sembilan for duration of at least 1 year. This was a cross sectional study design and parental informed consent was obtained prior to data collection. This study followed the tenets of the Declaration of Helsinki and received approval from Universiti Kebangsaan Malaysia Research Ethics Committee UKM 1.5.3.5/244/SPP2. Parental informed consent was obtained prior to data collection. The inclusion criteria was that the subjects are to be Orang Asli children aged between 7 to 12 years old. Whereas the exclusion criteria was Orang Asli children aged 13 years old and older. The Orang Asli School children were divided into two groups in accordance with age stratification practices of the Ministry Education of Malaysia i.e. Level 1: Aged from 7 to 9 years old, i.e. Standard 1 to 3 and Level 2: Aged from 10 to 12 years old i.e. Standard 4 to 6. The groupings illustrate the different levels of the syllabi delivered to Malaysian school-going children. It also reflects the differing levels of word difficulty used with the children. These children underwent the following visual screening; monocular visual acuity test (VA) using the Lea Symbol LogMAR Chart for distance; cover test (CT), Hirschberg’s test, ocular external assessment, ophthalmoscopy and refraction by Optometrists. Children who failed the vision screening were referred for further eye examination to confirm the diagnosis by an Ophthalmologist. The visual acuity of Orang Asli children who were prescribed glasses were retested at 6 weeks and were then diagnosed to have refractive amblyopia if their visual acuity was not able to achieve 6/6 monocularly. Table 1 shows the fail score criteria for each screening test [20]. All data obtained were analysed using the Statistical Package for Social Sciences (SPSS) version 19.0 software. Descriptive and frequency test applications are used to analyse the data to obtain mean values, standard deviations, median, range and percentages.

**Table 1:** Fail score criteria for each screening test

Test	Fail Score Criteria
Visual Acuity	< 0.30 LogMAR
Cover Test	Any obvious deviation to eye movement (tropia)
Hirschberg’s Test	Any decentration from corneal reflex
External Observation	Any abnormality
Ophthalmoscopy	Any abnormality
Cycloplegic Refraction	Myopia: > −0.50 D
	Hyperopia: > + 1.50 D
	Astigmatism: > 0.75 D
Prism Cover Test	Esophoria Near: > 5Δ Far: > 6Δ
	Exophoria Near: > 5Δ Far: > 10Δ
	Hyperphoria: > 2Δ
	Heterotropia: Any amount of heterotropia

Sources: Duratul et al. (2009b) [20]

## Results

**Fig. 1:**
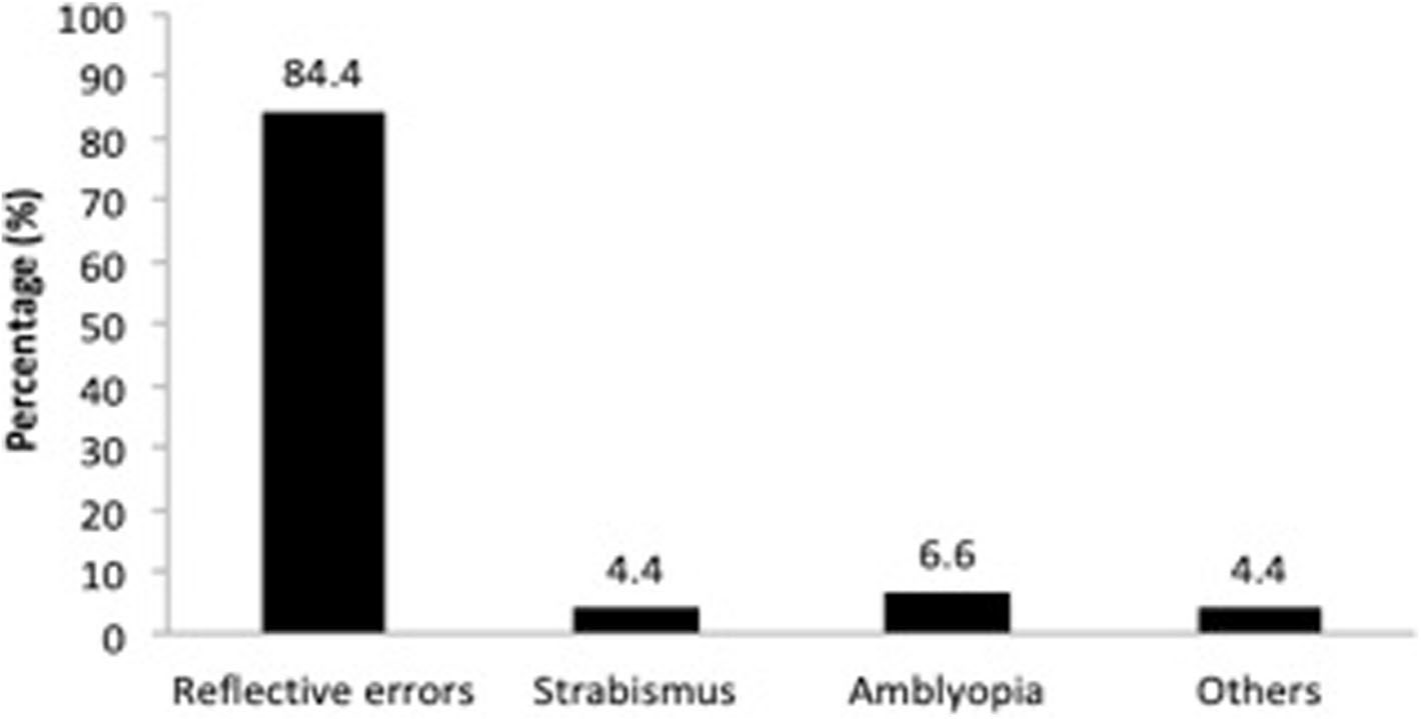
Distribution of Refractive Errors, Strabismus, Amblyopia and Others Ocular findings among Orang Asli Children

All the school children who participated in this study had never underwent vision screening previously nor had used any spectacles. 51% of them were boys while the remainders were girls. Of the 110 children who participated, 46 failed the vision screening and were referred to Pusat Perubatan Canselor Tuanku Mukhriz, Universiti Kebangsaan Malaysia for further examination. After further assessment of these 46 children, it was found that 45 children (40.9% of the total subject) were true positives while only 1 child was found to be a false positive for vision impairment. Of the 45 Orang Asli children majority of them were diagnosed with refractive errors followed by amblyopia, strabismus and others ocular findings (Fig. 1).

### Refractive errors and amblyopia

Thirty eight children were found to have refractive errors. For Level 1 age group, i.e. 7 to 9 years old, the mean spherical equivalent (SE) for RE was + 0.55 ± 0.63 (range − 1.00 D up to + 2.25 D) and for LE was + 0.54 ± 0.56 (range − 1.00 D up to + 1.75 D). For the Level 2 age group, 10 to 12 years old, the mean spherical equivalent (SE) for RE was + 0.27 ± 0.71 (range − 2.00 D up to + 1.75 D) and LE was + 0.31 ± 0.65 (range − 1.75 D up to + 1.75 D). Table 2 shows the spherical equivalent of the refractive errors. Correlation analysis on the age groups and on the SE of RE and LE was done. The results showed that there were significant relationships between the age groups and the SE (*p* = 0.005) for hyperopia. In this study, the ammetropia distribution pattern for refractive errors leaned towards hyperopia. A total of 31 (28.2%) children with almost equal number of female and male were found to have hyperopia. Six Orang Asli children had myopia and female had twice more incident of myopia compared to male Orang Asli children. Only one (0.9%) Orang Asli children had anti-metropia from the Level 2 age group. After using spectacles for 6 weeks, 3 (2.7%) children were not able to achieve a visual acuity of 6/6 monocularly and were diagnosed to have refractive amblyopia. Table 3 shows the distribution of types of refractive errors, amblyopia, strabismus and ocular abnormality among Orang Asli children.

**Table 2:** Spherical Equivalent and Range of Power of the Orang Asli Children Refractive Errors

Spherical Equivalent (SE)		Right Eye (Ds)/Power Range (Ds)	Left Eye (Ds)/Power Range (Ds)
Age	Level 1 (7–9 years old)	+ 0.55 ± 0.63/(−1.00, + 2.25)	+ 0.54 ± 0.56/(− 1.00, + 1.75)
	Level 2 (10–12 years old)	+ 0.27 ± 0.71/(− 2.00, + 1.75)	+ 0.31 ± 0.65/(− 1.75, + 1.75)

**Table 3:** Distribution of Types of Refractive Errors, Spherical Equivalent, Amblyopia, Strabismus and Ocular Abnormality among Orang Asli Children

Charateristcis		Types of Refractive Errors Number (%)				Amblyopia (*n* = 3)	Strabismus (*n* = 2)	Ocular abnormality (*n* = 2)
		Emmetropia (*n* = 72)	Myopia (*n* = 6)	Hyperopia (*n* = 31)	Anti-metropia (*n* = 1)			
Gender	Male	40 (36.4)	2 (1.8)	14 (12.7)	0 (0.0)	2 (1.8)	2 (1.8)	2 (1.8)
	Female	32 (29.1)	4 (3.6)	17 (15.5)	1 (0.9)	1 (0.9)	0 (0.0)	0 (0.0)
Age	Level 1 (7–9 years old)	34 (30.9)	1 (0.9)	23 (20.9)	0 (0)	3 (2.7)	2 (1.8)	2 (1.8)
	Level 2 (10–12 years old)	38 (34.5)	5 (4.5)	8 (7.3)	1 (0.9)	0 (0)	0 (0.0)	0 (0.0)

### Strabismus

Three children failed the Cover Test and Hirschberg’s Test during vision screening and were referred for further examination by an Ophthalmologist. After this examination, the children were found to have strabismus i.e. exotropia at 6 m fixation and were from the Level 1 age group (7 until 9 years old). Two of these children were male. Thus, the strabismus prevalence (all causes) among Orang Asli children in this study was 1.8% (Table 3).

### Ocular abnormality

Ocular abnormalities are all abnormal findings other than those mentioned earlier and include any structural or anatomical abnormalities of the eye. The prevalence of ocular abnormalities for this study was 1.8% i.e. involving two children. Both these children were male and one of them, aged 7 years old, was found to have a high cup-to disc ratio while the other had anisocoria (unequal pupil size) between his two eyes (Table 3).

## Discussion

In general, the causes of vision impairment among Orang Asli children in this study included refractive errors, amblyopia, strabismus and ocular abnormalities. These findings were similar to the study conducted by Zainal et al., (2002) [21]. However, there is some difficulty in discussing the causes of vision impairment among Orang Asli children because there have been limited vision status or profile studies conducted in Malaysia. Refractive errors were the major cause of vision impairment (34.5%) among Orang Asli children. This is in agreement with the findings of The National Eye Survey 1996 which identified 48% of Malaysians, especially children aged 7 years and above, have uncorrected refractive errors [21].

In this study involving Orang Asli of the Temuan tribe, hyperopia was the leading cause (28.2%) of refractive errors followed by myopia (5.5%). Further analysis showed that there was a significant correlation between SE refractive errors with age for hyperopia. It was also noted that the descriptive data showed an increase in percentage of refractive errors as age increases for hyperopia. This is in contrast to other studies conducted among other Malaysian ethnicities [22–24] such as Malay, Chinese or Indian children, in which myopia was the leading cause of refractive errors, where these errors ranged from 4% [14] to 17.1% [25], these studies being conducted on preschool and secondary school children respectively.

Amblyopia was also another cause of vision impairment among these Orang Asli children, and if undetected and corrected could affected the children ability to do well in school. Amblyopia is a condition where visual acuity is worse than 6/6 without the presence of any organic cause or ocular pathology [26]. The prevalence of amblyopia among these Orang Asli children was 2.7%. This is in agreement with a study conducted among school children aged 7 years and above, in the Gombak District in Malaysia where they found the prevalence was 3% [25]. Our findings were higher when compared to other studies conducted among school children in Singapore, South Korea and Netherlands which ranges from 0.4 to 2% [27–30]. Holmes & Clarke recommended that early stage intervention should be provided for children with amblyopia, ideally before the age of 8 years old so that they would have better prognosis [26]. Where possible, screening should be done as early as possible, at between 2 to 4 years old, to provide the best visual recovery.

The prevalence of strabismus and ocular abnormalities was found to be 1.8 and 2.7% respectively in this study. The prevalence pattern for these impairments was similar to that found in most countries. This is because strabismus and ocular abnormalities are quite easily identified by parents in the early stages because the impairment has clear signs and symptoms. Therefore, parents often seek treatment for the problem as soon they notice it.

Understanding and determining the prevalence and causes of vision impairment among Orang Asli school children will provide a clearer picture of their visual status needs. Hence proper and early intervention should be provided in the medical health services provided to the Orang Asli community especially with regards vision care. The visual impairment issues particularly refractive errors can be easily addressed and thus avoidable blindness can then be easily rectified.

## Conclusion

The prevalence of visual impairment among Orang Asli children in this study was 34.5% where the main cause was refractive errors. The main cause of refractive errors found to be hyperopia (28.2%) followed by amblyopia (2.7%), strabismus (1.8%) and ocular abnormalities (1.8%). Hyperopia, which is associated with symptoms such as asthenopia, frontal headache and blurred vision at near tasks among children, may results in these children avoiding near work such as writing or reading. Thus, vision screening and a comprehensive eye examination is very important and needs to be done on all Orang Asli children so that any visual problems can be detected at an early stage to avoid developmental learning difficulties among these already disadvantaged children.

## Abbreviations

CT: Cover test
LE: Left eye
RE: Right eye
SPSS: Statistical Package for Social Sciences
VA: Visual acuity
